# Supplementary material for: A Mechanistic Link from GABA to Cortical Architecture and Perception
Source: Curr Biol. 2017 Jun 5;27(11):1685–1691.e3. doi: 10.1016/j.cub.2017.04.055 (PMC5462622; doi:10.1016/j.cub.2017.04.055)
Supplement: Document S1. Figures S1–S4 and Tables S1–S4 [file mmc1.pdf]

Current Biology, Volume *27*

Supplemental Information

A Mechanistic Link from GABA

to Cortical Architecture and Perception

James Kolasinski, John P. Logan, Emily L. Hinson, Daniel Manners, Amir P. Divanbeighi Zand, Tamar R. Makin, Uzay E. Emir, and Charlotte J. Stagg

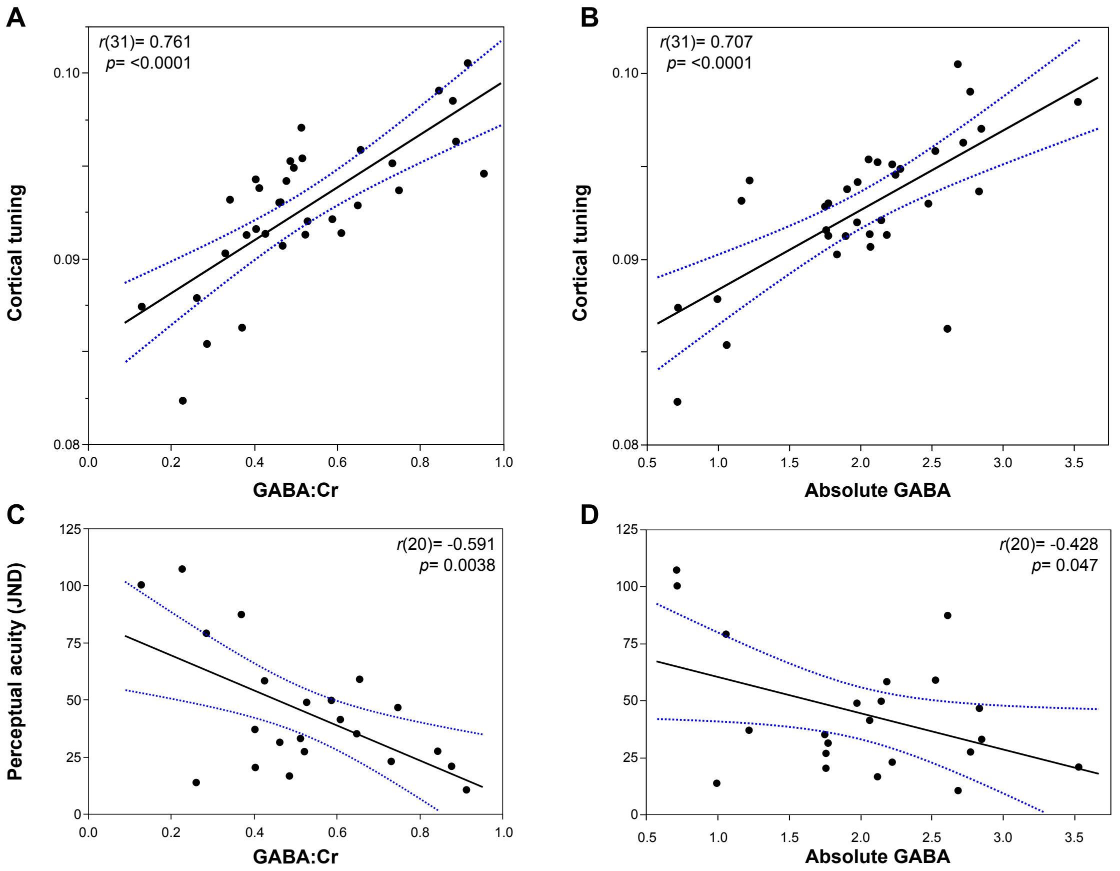

Figure S1. Correlations with GABA:Cr persist for raw GABA concentrations. Related to Figures 2 & 3. The observed patterns of correlation between normalised GABA:Cr values and both cortical tuning (A) and perceptual acuity (C) were also observed for values of absolute GABA quantified directly from the LCmodel MRS data analysis (B/D). In this combined cohort, the relationship between cortical tuning and tactile perceptual acuity was also still observed: r(20)=-0.734 p=0.0001 (not shown).

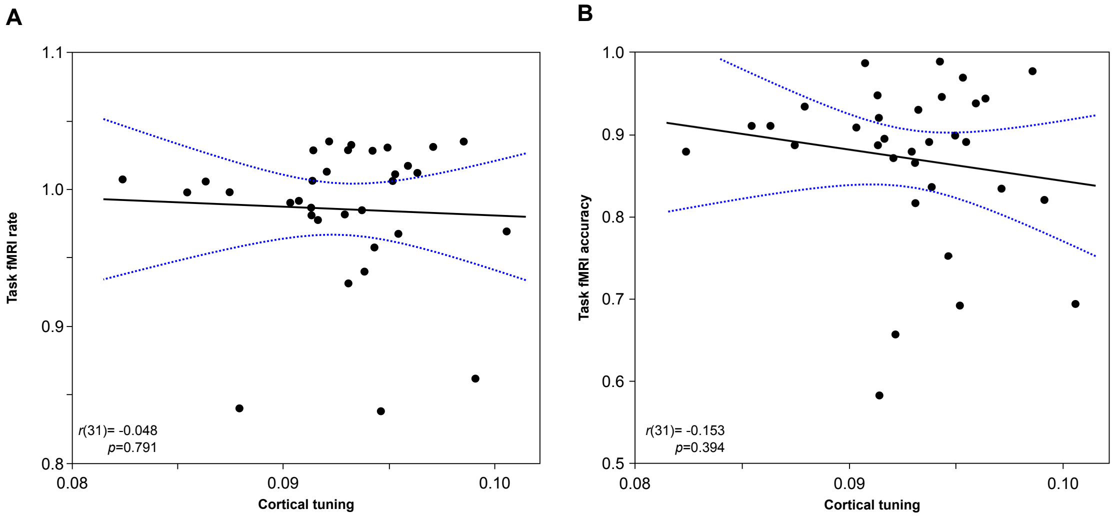

Figure S2. Cortical tuning metrics calculated in S1 show no relationship with motor performance during fMRI task. Related to Figures 2 & 3. Neither task fMRI motor rate (A: button presses per second) nor accuracy (B: percentage correct button presses) correlated with the magnitude of the cortical tuning metric across participants.

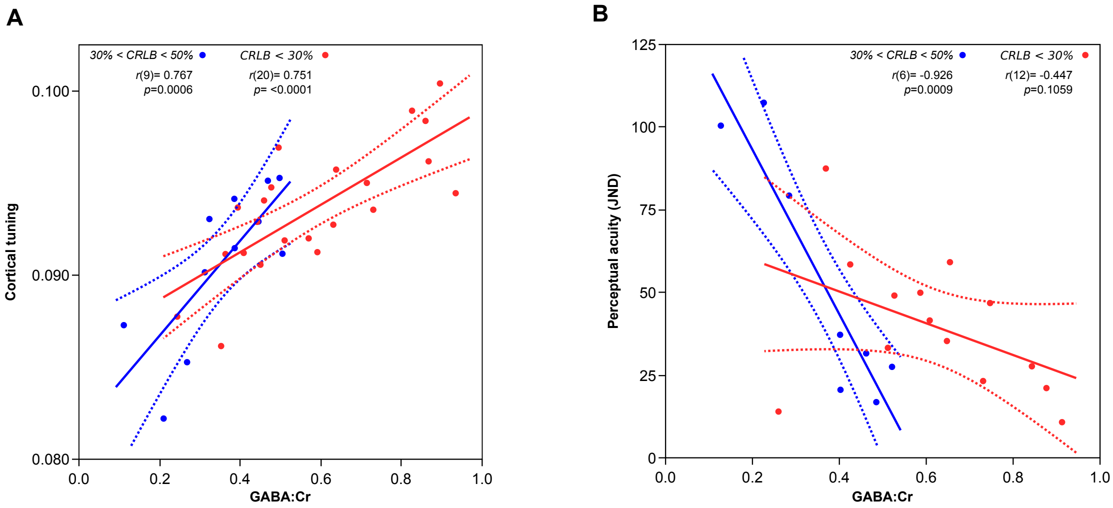

Figure S3. Analysis of correlations of GABAergic tone metrics assessed by MRS model fit. Related to Figures 2 & 3. Correlations between GABA:Cr and both cortical tuning and tactile perceptual acuity are present across the range of CRLB thresholds applied to fitted MRS data.

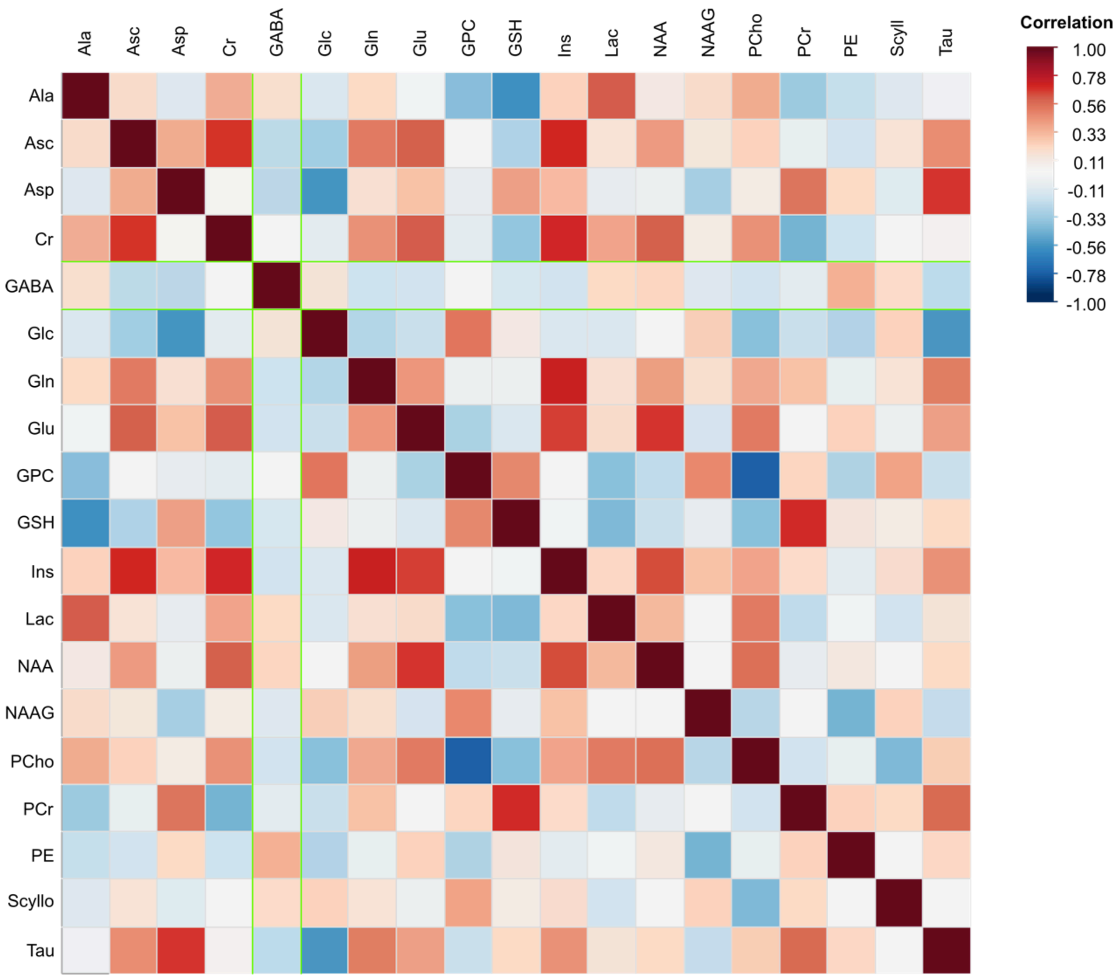

Figure S4. Cross-correlation of metabolites quantified by MRS in all participants. Related to Figures 2 & 3. Pearson's correlation coefficient shows no correlations between absolute concentrations of GABA and other metabolites (green column/row) greater in magnitude than +/- 0.35. This result indicates good spectral separation of GABA.

		ID	Age	Gender	Handedness
**Experiment one**	*MR only*	1-001	23	F	R
		1-002	26	F	R
		1-003	28	F	R
		1-004	25	M	R
		1-005	20	M	R
		1-006	20	M	R
		1-007	19	M	R
		1-008	22	M	R
		1-009	32	F	R
		1-010	29	M	R
		1-011	20	F	R
	MR + behaviour	1-012	21	M	R
		1-013	20	M	R
		1-014	30	F	R
		1-015	21	M	R
		1-016	24	F	R
		1-017	21	M	R
		1-018	20	M	R
		1-019	20	M	R
		1-020	20	F	R
		1-021	21	F	R
		1-022	21	F	R
**Experiment two**	*MR + behaviour*	2-001	20	F	R
		2-002	26	M	R
		2-003	27	F	R
		2-004	20	F	R
		2-005	20	M	R
		2-006	22	M	R
		2-007	20	M	R
		2-008	20	F	R
		2-009	19	F	R
		2-010	21	M	R
		2-011	20	M	R

Table S1. Demographic information for participants recruited to fMRI and behavioural cohorts. Related to Figure 1. F: female, M: Male, R: right handed, MR: magnetic resonance.

ID	Tuning: time point 1	Tuning: time point 2
3-001	0.089074	0.090526
3-002	0.093360	0.090194
3-003	0.091256	0.090504
3-004	0.091828	0.090704
3-005	0.088730	0.091726
3-006	0.091622	0.088814
3-007	0.088088	0.087834
3-008	0.086516	0.087832
3-009	0.085434	0.083752
3-010	0.089918	0.089202
3-011	0.085746	0.084584
3-012	0.083318	0.084836
3-013	0.089762	0.091070
3-014	0.090726	0.090726
3-015	0.088142	0.088264
3-016	0.088274	0.088102

Table S2. Test-Retest reliability of cortical tuning metric. Related to Figures 2 & 3. Data collected during the same task fMRI paradigm in a cohort independent of the current study during two scan sessions, separated by 4 weeks. A high degree of internal consistency was observed between the JND measurements derived on separate days. The single measures intraclass correlation (ICC) was 0.802 (95% confidence interval: 0.520 - 0.926). ICC calculated using a two-way random effects model with absolute agreement.

ID	JND: time point 1	JND: time point 2
4-001	23.591	23.566
4-002	57.429	64.634
4-003	30.486	40.327
4-004	39.912	37.693
4-005	45.139	48.660
4-006	31.440	39.458
4-007	25.683	39.682
4-008	32.320	41.779
4-009	23.217	14.694
4-010	27.674	24.719
4-011	46.802	40.453
4-012	35.319	43.581
4-013	49.924	46.385
4-014	106.719	77.447
4-015	10.774	23.359
4-016	45.879	53.090

Table S3. Test-Retest reliability of JND measures. Related to Figures 2 & 3. Data collected during a temporal order judgment task in a cohort independent of the current study during two behavioural testing sessions, separated by 1 week. A high degree of internal consistency was observed between the JND measurements derived on separate days. The single measures intraclass correlation (ICC) was 0.842 (95% confidence interval: 0.610 - 0.942). ICC calculated using a two-way random effects model with absolute agreement. JND: Just Noticeable Difference

		Consequent						
		M (Tuning)				Y (JND)		
Antecedent		Coeff.	*SE*	*p*		Coeff.	*SE*	*p*
X (GABA)	*a*	0.1807	0.0259	<0.001	*c'*	-0.0118	0.0373	0.7552
M (Tuning)		-	-	-	*b*	0.4893	0.1739	0.0110
Constant	i_1	0.8298	0.0147	<0.001	i_2	-0.2909	0.1447	0.0588
		R^2= 0.7086<br>$F_{(1,20)}$=48.6356 p<0.001				R^2= 0.5409<br>$F_{(2,19)}$=11.1908 p<0.001		
		Total effect (*c*)<br>Coeff.= 0.0766 SE= 0.0234<br>p=0.0038<br><br>Direct effect (*c'*)<br>Coeff.= -0.0118 SE= 0.0373<br>p=0.7552				Indirect effect (ab)<br>Coeff.= 0.0884 SE=0.0359<br><br>Preacher and Kelley κ^2<br>κ^2=0.46 SE=0.1479		

Table S4. Summary statistics from mediation analysis. Related to Figure 4.
