# Supplementary material for: A Mechanistic Link from GABA to Cortical Architecture and Perception
Source: Curr Biol. 2017 Jun 5;27(11):1685–1691.e3. doi: 10.1016/j.cub.2017.04.055 (PMC5462622; doi:10.1016/j.cub.2017.04.055)
Supplement: Document S2. Article plus Supplemental Information [file mmc2.pdf]

Report

Current Biology

A Mechanistic Link from GABA to Cortical Architecture and Perception

Graphical Abstract

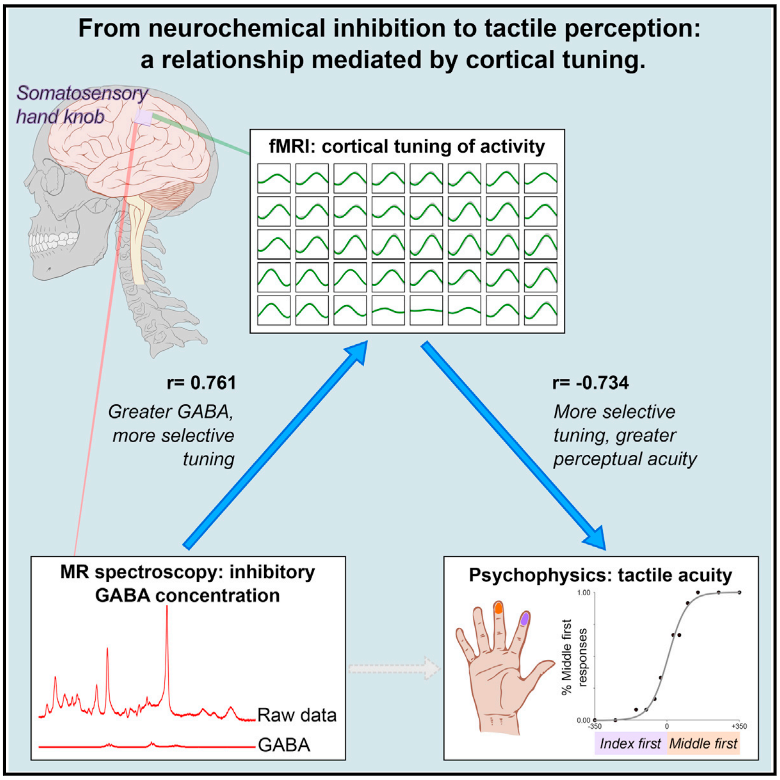

Highlights

- GABAergic tone correlates with perceptual acuity in the human somatosensory system
- This relationship is mediated by the tuning of activity in somatosensory cortex
- We explain perceptual acuity via the underlying cellular and metabolic processes

Authors

James Kolasinski, John P. Logan, Emily L. Hinson, ..., Tamar R. Makin, Uzay E. Emir, Charlotte J. Stagg

Correspondence

james.kolasinski@ndcn.ox.ac.uk

In Brief

γ-aminobutyric acid (GABA) is implicated in the tuning of activity in the primary sensory cortices. Kolasinski et al. demonstrate that individual differences in human perceptual acuity can be explained by differences in cortical GABAergic tone, a relationship mediated by differences in the tuning of cortical activity.

Kolasinski et al., 2017, Current Biology *27*, 1685–1691
June 5, 2017 © 2017 The Author(s). Published by Elsevier Ltd.
http://dx.doi.org/10.1016/j.cub.2017.04.055

Current Biology
Report

CellPress

A Mechanistic Link from GABA to Cortical Architecture and Perception

James Kolasinski,[1,2,3,6,*] John P. Logan,[1] Emily L. Hinson,[1,4] Daniel Manners,[1] Amir P. Divanbeighi Zand,[1] Tamar R. Makin,[1,5] Uzay E. Emir,[1] and Charlotte J. Stagg[1,4]
[1]Oxford Centre for fMRI of the Brain, Nuffield Department of Clinical Neurosciences, University of Oxford, Oxford OX3 9DU, UK
[2]Cardiff University Brain Research Imaging Centre, School of Psychology, Cardiff University, Cardiff CF24 4HQ, UK
[3]University College, Oxford OX1 4BH, UK
[4]Oxford Centre for Human Brain Activity, Department of Psychiatry, University of Oxford, Oxford OX3 7JX, UK
[5]Institute of Cognitive Neuroscience, University College London, London WC1N 3AZ, UK
[6]Lead Contact
*Correspondence: james.kolasinski@ndcn.ox.ac.uk
http://dx.doi.org/10.1016/j.cub.2017.04.055

SUMMARY

Understanding both the organization of the human cortex and its relation to the performance of distinct functions is fundamental in neuroscience. The primary sensory cortices display topographic organization, whereby receptive fields follow a characteristic pattern, from tonotopy to retinotopy to somatotopy [1]. GABAergic signaling is vital to the maintenance of cortical receptive fields [2]; however, it is unclear how this fine-grain inhibition relates to measurable patterns of perception [3, 4]. Based on perceptual changes following perturbation of the GABAergic system, it is conceivable that the resting level of cortical GABAergic tone directly relates to the spatial specificity of activation in response to a given input [5–7]. The specificity of cortical activation can be considered in terms of cortical tuning: greater cortical tuning yields more localized recruitment of cortical territory in response to a given input. We applied a combination of fMRI, MR spectroscopy, and psychophysics to substantiate the link between the cortical neurochemical milieu, the tuning of cortical activity, and variability in perceptual acuity, using human somatosensory cortex as a model. We provide data that explain human perceptual acuity in terms of both the underlying cellular and metabolic processes. Specifically, higher concentrations of sensorimotor GABA are associated with more selective cortical tuning, which in turn is associated with enhanced perception. These results show anatomical and neurochemical specificity and are replicated in an independent cohort. The mechanistic link from neurochemistry to perception provides a vital step in understanding population variability in sensory behavior, informing metabolic therapeutic interventions to restore perceptual abilities clinically.

RESULTS

We hypothesized that extra-synaptic GABAergic tone acts to localize such cortical activation, with greater GABA concentrations leading to more selective cortical tuning, resulting in greater perceptual acuity. To test this hypothesis, we investigated the relationship between the concentration of cortical GABA and neuronal tuning in human primary somatosensory cortex (S1), relating these to tactile perceptual acuity. In experiment 1, we used a combination of fMRI and magnetic resonance spectroscopy (MRS) at 7 T to acquire measurements in the left-hand knob region of human S1 (Figure 1A). Cortical tuning was quantified using an fMRI task involving a continuous cycle of movement of individual fingers of the right hand, with no rest periods, implemented in a well-validated "traveling-wave" analysis [8] (Figure 1B). For voxels in the anatomical hand knob, the spread of activation across this cycle was quantified as a measure of cortical tuning, where higher tuning is associated with a sharper and more selective response profile during the movement cycle (Figure 1C). MRS was used to quantify levels of sensorimotor cortical GABA and glutamate (Figure 1D). Measures of perceptual acuity were acquired using a temporal order judgment task, involving the delivery of variably spaced pairs of vibrotactile stimuli, one each to the index and middle finger of the right hand; the resulting accuracy data were used to calculate a measure of perceptual acuity (Figure 1E).

Inhibition, Tuning, and Perceptual Acuity

There was a strong positive correlation between cortical tuning in S1 and resting S1 GABA ($r = 0.676$, $n = 22$, $p = 0.0006$); higher levels of cortical GABAergic tone were associated with greater cortical tuning (Figure 2A). This relationship was not observed between S1 cortical tuning and S1 glutamate ($r = 0.146$, $n = 22$, $p = 0.518$); these two correlations differed significantly ($Z = 2.45$, $p = 0.0071$) [9]. The same pattern of relationships was also present in the smaller cohort of 11 for whom measures of perceptual acuity were also acquired (S1 GABA versus S1 tuning: $r = 0.731$, $n = 11$, $p = 0.011$; S1 glutamate versus S1 cortical tuning: $r = -0.053$, $n = 11$, $p = 0.877$; Figure 2A: filled circles).

There was also a strong correlation between cortical tuning in S1 and perceptual acuity in the tactile temporal order judgment task ($r = -0.703$, $n = 11$, $p = 0.016$) (Figure 2C), suggesting that,

This is an open access article under the CC BY license (http://creativecommons.org/licenses/by/4.0/).

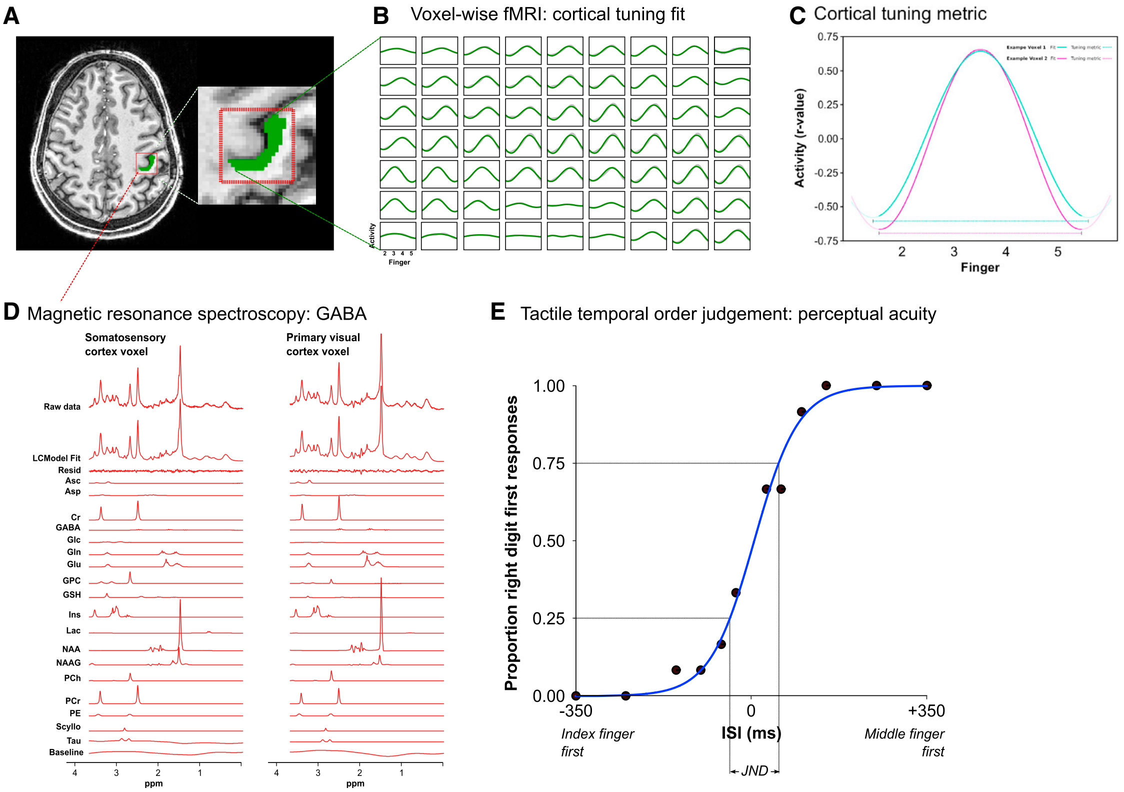

Figure 1. Measuring GABA and Cortical Tuning in Human Somatosensory Cortex and Their Relationship to Perceptual Acuity

(A) Representative participant: MRS data acquired in sensorimotor cortex (red voxel: 2 × 2 × 2cm) centered on the hand knob and primary visual cortex (experiment 2 only). Thresholded fMRI activation, green.

(B) fMRI voxel-wise cortical tuning profile of somatosensory cortex activity across the movement cycle (gray) from index finger (2) to little finger (5), fitted with a sinusoid (green): sample of adjacent voxels from one fMRI acquisition in one participant.

(C) Using the sinusoid fit to the cortical tuning profile during the cycle of digit movement (solid lines), a cortical tuning metric reflecting the selectivity of each voxel's activity to a specific digit was defined as the period of the sinusoid fit (dashed lines), expressed as the inverse (1/period).

(D) Representative MRS spectra from one participant.

(E) Tactile temporal order judgment task quantifying perceptual acuity: representative accuracy data from one run/participant. ISI, interstimulus interval. Participant demographic information is provided in Table S1.

as hypothesized, more selective activation of S1 is associated with better performance in terms of tactile perception.

Furthermore, a strong correlation was observed between S1 GABAergic tone and tactile perceptual acuity ($r = -0.687$, $n = 11$, $p = 0.02$) (Figure 2B), which does not generalize to the other major cortical neurotransmitter glutamate ($r = -0.182$, $n = 11$, $p = 0.592$). These results demonstrate that higher levels of cortical GABA, but not glutamate, are associated with enhanced tactile function.

To formally test the hypothesis that measures of GABA but not glutamate correlate with cortical tuning and perceptual acuity, we used distinct procedures to detect any significant (unpredicted) glutamatergic correlations, and to ensure consistent (predicted) GABAergic correlations in the cohort for whom all data were present in experiment 1 ($n = 11$). Specifically, we apply a conjunction test over all GABAergic (predicted) correlations and an omnibus test over all glutamatergic correlations [10]. In this stringent analysis, the compound null hypothesis is disproved only if all GABAergic correlations are significant in the absence of significant glutamatergic correlations. The conjunction test therefore considers the maximum p value across all GABAergic correlations ($p = 0.02$), while the omnibus test considers the minimum p value from all glutamatergic correlations ($p = 0.592$), the combination of which provide evidence to reject the null hypothesis. This liberal test offers good power to detect any possible correlations that deviate from the expected hypothesis and to account for multiple correlation analyses undertaken.

Replication and Anatomical Specificity

The pattern of correlation between fMRI cortical tuning, MRS-derived GABAergic tone, and tactile perceptual acuity observed in experiment 1 was replicated in an entirely new cohort in experiment 2 (Figure 3; $n = 11$). Similarly, the combination of a conjunction and omnibus test formally demonstrated once again that GABA but not glutamate correlates with tactile perceptual acuity and cortical tuning (conjunction test maximum p value across all GABAergic correlations: $p = 0.024$; omnibus test minimum p value across all glutamatergic correlations: $p = 0.259$). Furthermore, in experiment 2, an additional control MRS voxel was acquired in primary visual cortex (V1, $n = 10$), centered

CellPress

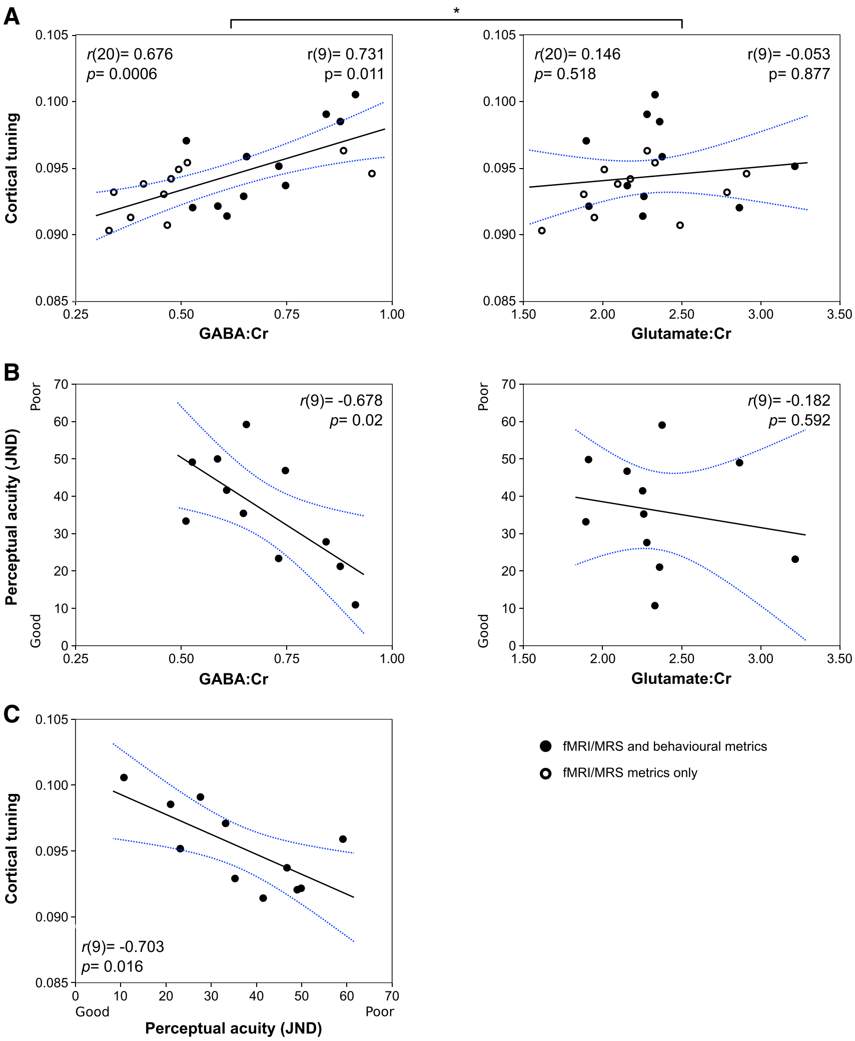

Figure 2. GABAergic Tone Reflects Cortical Tuning and Tactile Perceptual Acuity in the Somatosensory System: Experiment 1

(A) A strong relationship exists between cortical tuning in S1 and local GABA, but not glutamate.

(B) Tactile perceptual acuity correlates strongly with S1 GABA, but not with S1 glutamate.

(C) Cortical tuning correlates with tactile perceptual acuity; greater tuning is associated with better tactile abilities. *p < 0.05 Hittner's test. JND, just noticeable difference; Cr, total creatine. Blue lines, confidence bounds. The significant difference in (A) persists with n = 11 (filled circles; Hittner's $Z = 2.00$, p = 0.023).

See also Figures S1–S4 and Tables S2 and S3.

bilaterally over the calcarine sulcus of the occipital cortex. The observed relationships of GABA levels in S1 did not persist for GABA levels measured in V1 (Figures 3A and 3B). The combination of a conjunction and omnibus test formally appraises the anatomical specificity of the relationship of S1 GABA but not V1 GABA with tactile perceptual acuity and cortical tuning (conjunction test maximum p value across all S1 GABAergic correlations: p = 0.024; omnibus test minimum p value across all V1 GABAergic correlations: p = 0.548). This demonstrates the anatomical specificity in the observed pattern of the correlations between S1 GABA and tuning seen in experiments 1 and 2.

In summary, cortical GABAergic tone in S1 shows both anatomical and neurochemical specificity in its relationships with local cortical tuning and measures of tactile perceptual acuity. In light of the link between cortical GABA and glutamate, both in terms of excitatory/inhibitory balance, and their shared metabolic pathways, the correlations involving GABA:Cr levels were also computed as partial correlations correcting for Glutamate:Cr: these correlations remain significant ($p < 0.05$) and do not differ qualitatively from those presented in Figures 2 and 3. The observed correlations of GABA:Cr also held for values of raw GABA concentration (Figure S1). The reproducibility of the cortical tuning metric and just noticeable difference (JND) have been validated in two independent cohorts, each of 16 participants, for whom these measures were calculated across two different time points using a single-measures intraclass correlation (ICC; two-way random effects model with absolute agreement). Cortical tuning ICC: 0.802 (Table S2; 95% CI: 0.520–0.926); JND ICC: 0.842 (Table S3; 95% CI: 0.610–0.942).

Mediation Analysis

In order to more fully investigate the potential role of cortical tuning in S1 as a mechanistic link between S1 inhibitory GABAergic tone and tactile perceptual acuity, a mediation analysis [11] was applied to the MR and behavioral data pooled from experiments 1 and 2 (n = 22). Mediation approaches assess the mechanism through which two variables are related. Mediation is well suited when the mediator (M_i) is the logical effect of one variable (X), and the logical cause of another variable (Y). In this study, cortical tuning (M_i) is the logical effect of inhibitory GABAergic tone (X: GABA:Cr) and the logical cause of differences in tactile perceptual acuity (Y: JND) (Figure 4). Mediation was conducted using regression with bootstrapping to ascertain whether cortical tuning accounts for the link between cortical inhibition and perceptual abilities (Figure 4A).

There was strong evidence that cortical tuning (M_i) completely mediates the link between GABA:Cr (X) and perceptual acuity (Y), with a percentage mediation (P_M) of 88% (Figure 4B). This resulted from a significant indirect effect (c) of greater magnitude than the direct effect (c′), which itself was not significant (Table S4). The significance of this mediation effect is further demonstrated by the Sobel test ($Z = 2.59$, p = 0.0097) and a Preacher and Kelley $\kappa^2 = 0.46$. These results implicate the tuning response of cortical neurons as a possible mechanism via which the observed relationship between variability in the inhibitory neurochemical milieu and perceptual abilities may be effected (Figures 2 and 3).

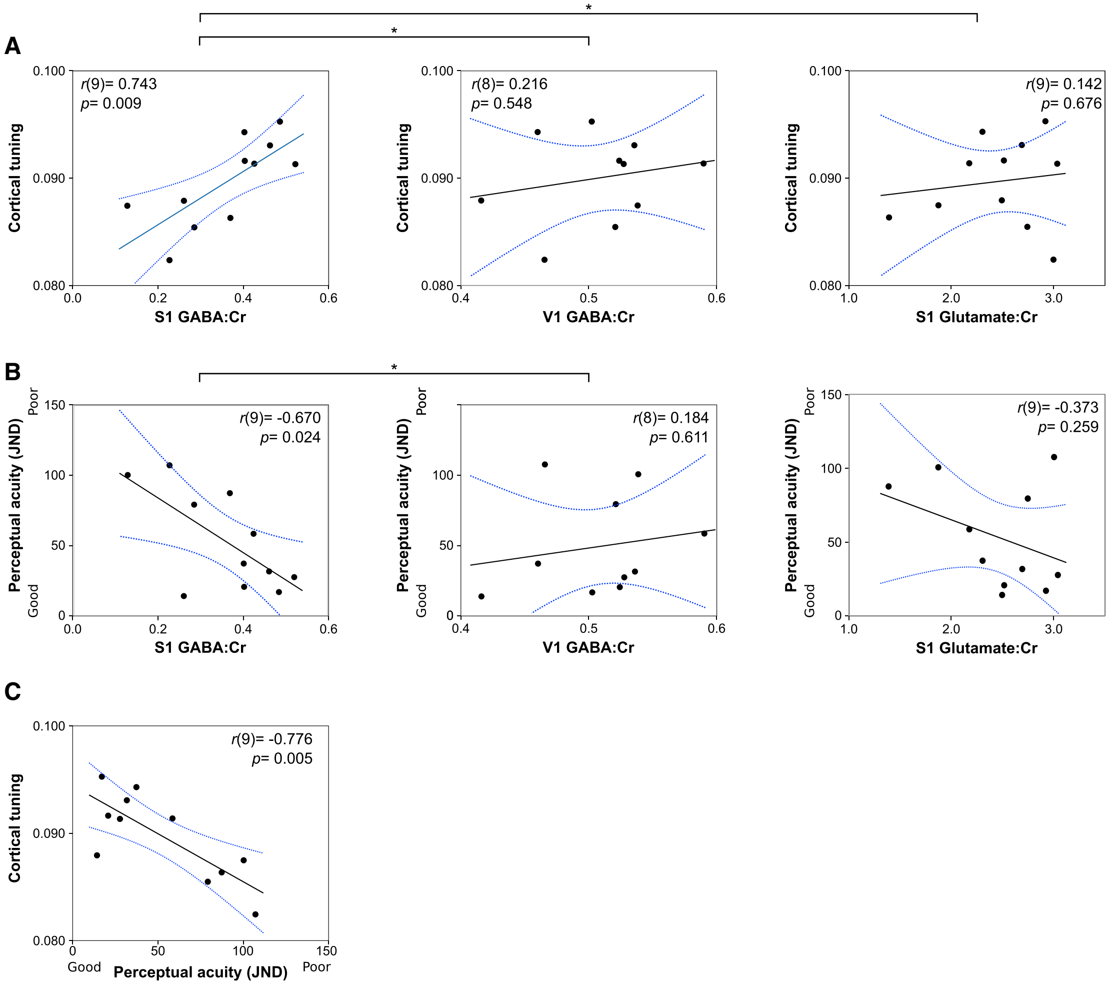

Figure 3. GABAergic Relationships Show Anatomical Specificity: Experiment 2

An independent replication of the correlations between S1 GABAergic tone, cortical tuning, and tactile perceptual acuity in experiment 1, and evidence of the anatomical specificity of the neurochemical relationship using a V1 MRS control voxel.

(A) Significant correlations between cortical tuning in S1 and S1 cortical GABA, but neither S1 glutamate (Hittner's: $Z = 2.60$, $p = 0.0047$) nor V1 GABA (Hittner's $Z = 1.65$, $p = 0.049$).

(B) Tactile perceptual acuity correlates strongly with S1 GABA, but with neither S1 glutamate, nor V1 GABA (Hittner's $Z = -2.31$, $p = 0.0104$).

(C) Cortical tuning correlates with tactile perceptual acuity. *$p < 0.05$ Hittner's test. Abbreviations as per Figure 2.

See also Figures S1–S4 and Tables S2 and S3.

DISCUSSION

These data demonstrate for the first time the ability to explain perceptual acuity in terms of both the underlying cellular and metabolic processes in the human brain. That is to say, those individuals with greater tactile perceptual abilities show more selective cortical activity in primary somatosensory cortex, underpinned by greater local concentrations of inhibitory GABA, which acts to localize the spread of excitatory signaling, maintaining the functional architecture of the cortex.

The relationship between inhibition and cortical tuning could result from two related forms of GABAergic signaling. In addition to phasic synaptic GABAergic activity, extracellular GABA also has a neuromodulatory role via tonic signaling on extra-synaptic $GABA_A$ receptors [12, 13]. This extra-synaptic GABAergic "tone" drives a persistent ambient level of inhibition, affecting neuronal excitability distinct from the time-dependent phasic inhibitory signaling that underpins lateral inhibition, including changes to the membrane refractory period [14, 15]. Both tonic and phasic GABAergic activity shape the selective response profiles of neurons in the primary sensory regions in the brain [16], yielding the topographic organization vital to sensory perception [17].

MRS GABA signals more closely reflect extra-synaptic GABAergic tone rather than phasic synaptic GABA signaling, due to interactions of vesicular GABA with macromolecules [18]. Modeling of the neuromodulatory effects of tonic inhibition has investigated the impact of an accumulation of extra-synaptic GABA on the selective response profiles of cortical columns, which underpins cortical tuning [19]. This work suggests that following phasic GABAergic signaling in a specific stimulus-relevant column, the spillover and diffusion of cortical GABA leads to an increase in tonic inhibitory tone in the adjacent but

A

$$M = i_1 + aX + e_M$$

$$Y = i_2 + c'X + bM + e_Y$$

$$a = [\hat{M} | (X = x)] - [\hat{M} | (X = x - 1)]$$

$$b = [\hat{Y} | (M = m, X = x)] - [\hat{Y} | (M = m - 1, X = x)]$$

$$c' = [\hat{Y} | (X = x, M = m)] - [\hat{Y} | (X = x - 1, M = m)]$$

$$c = c' + ab$$

$$Sobel\ test: t = \frac{ab}{\sqrt{a^2\sigma_b^2 + b^2\sigma_a^2}}$$

$$\kappa^2 = \frac{ab}{\mathfrak{M}(ab)}$$

B

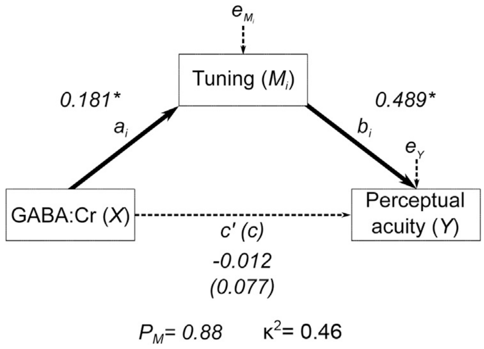

Figure 4. Cortical Tuning Exerts a Complete Mediating Effect on the Relationship between GABAergic Tone and Perceptual Acuity

MRS, fMRI, and behavioral data pooled from experiments 1 and 2 (n = 22).

(A) Mediation approach applied to investigate the link between GABA concentrations (X), cortical tuning (M_i), and perceptual acuity (Y).

(B) A significant mediating effect of cortical tuning (Mi) in the relationship between GABAergic tone and perceptual acuity. The strong mediating effect of cortical tuning accounted for a considerable proportion of the total relationship between GABA and perceptual acuity (PM = 0.88; Sobel test, $Z = 2.59$, $p = 0.0097$, Preacher and Kelley $\kappa^2 = 0.4651$.) For full mediation statistics, see also Table S4.

stimulus-irrelevant columns: a kind of non-synaptic lateral inhibition. Evidence for GABAergic spillover and non-synaptic signaling has been reported in various cortical and subcortical systems, including layer V pyramidal neurons in the somatosensory cortex [12, 20, 21].

Due to the size of MRS voxel used to measure S1 GABA, adjacent regions of motor cortex were also included in this measure of local inhibitory tone. The anatomical specificity of the observed relationships has been confirmed through the use of a control voxel in primary visual cortex (Figure 3). Moreover, reports of the regional GABA distributions in the primate cortex provide no strong evidence for local concentrations differences between the pre-central and post-central cortices [22].

Tonic GABAergic signaling clearly plays a key role in controlling the selective activation of cortical neuronal networks; however, additional factors also likely play a role in the maintenance of functional organization in the cortex. One candidate is variability in intra-cortical connectivity via so-called cortical sub-networks: inter-digitating networks of excitatory neurons largely, but not exclusively, tuned for similar sensory features [23]. Sub-networks amplify the cortical response to thalamocortical inputs across regions encoding specific sensory features [24]. By amplifying and prolonging sensory signaling in the cortex [25, 26], it is possible that these sub-networks effect more complex feature integration or perception. Therefore, while inhibitory tone localizes neuronal activity to ensure selective response profiles and specific cortical tuning, it is possible that an opposite factor of neuronal intracortical connectivity dictates the spread of excitation.

Although our results demonstrate that local cortical inhibitory tone and the selectivity of S1 activation were strongly related to tactile perceptual performance, neither sensorimotor GABA concentrations nor S1 cortical tuning were able to fully account for the variance in JND, reflecting performance in the temporal order judgment task. This is unsurprising, given that S1 acts as an early waypoint in a more distributed cortical network of tactile sensory perception, engaging parietal and frontal regions to integrate the spatial features of the stimuli [27], and inferring their relative timings in the multisensory temporal-parietal junction [28, 29].

The activation of S1 underlying the calculation of the cortical tuning metric was driven by naturalistic movement of the hand and therefore recruited a range of somaesthetic submodalities. While somatosensory processing has long been thought to segregate inputs from different varieties of cutaneous and proprioceptive receptors, more recent single-neuron recordings from non-human primates suggest a more integrative multimodal processing across all of the constituent areas of S1 [30, 31]. This integration of somaesthetic modalities is supported by the observation in this study that S1 cortical tuning responses elicited by movement are correlated with features of cortical structure inferred from sensory inputs alone in the measurement of perceptual acuity [30]. It is not possible to accurately define the boundaries of S1 Brodmann areas from gross anatomy for further analysis, the tactile perception task applied here will likely recruit neuronal populations across these traditional boundaries [31, 32].

The fMRI task data from which the cortical tuning metric was derived involved movement of individual fingers. The observed S1 activity was therefore likely composed of both ascending somatosensory afferents from the hand, as well as efference information via cortico-cortical connections with primary motor cortex (M1). Although it is thought that corollary activity from M1 might mimic the sensory feedback expected from a given movement [33], such predictive top-down control has not been directly reported in human S1. Recent evidence in rodent studies has provided strong evidence for disinhibitory connections between M1 and S1 that disinhibit topographically relevant regions of sensory cortex prior to any tactile stimulation [34, 35]. Importantly, no relationship was observed between the rate or accuracy of finger movements during the fMRI task and the cortical tuning metric (Figure S2). Moreover, the extent of finger movement individualization is unlikely to have driven variability in the cortical tuning curves. While anatomical enslavement in the hand affects individual finger movements at higher angular flexion, detailed hand kinematics data have demonstrated that, for low angular flexion of individual fingers, analogous to those used for button presses during the fMRI tasks, a high degree of independent finger movement is possible in the human hand [36].

The results presented here provide, for the first time, a direct link from neurochemicals at the metabolic level, to cortical responses in neuronal architecture, and on to perceptual abilities at the level of human behavior. This provides a fundamental advance in understanding how functional cortical organization is maintained and maps to perception. This insight offers a vital first step in the targeted development of clinical therapeutic strategies to modulate the cortical neurochemical milieu, in

order to effect selective changes in neuronal signaling and behavior.

STAR★METHODS

Detailed methods are provided in the online version of this paper and include the following:

- KEY RESOURCES TABLE
- CONTACT FOR RESOURCE SHARING
- EXPERIMENTAL SUBJECT DETAILS
- METHOD DETAILS
  - MR acquisition
  - fMRI task
  - fMRI analysis
  - MRS analysis
  - Behavioral data acquisition
  - Behavioral data analysis
  - Reproducibility measures
- QUANTIFICATION AND STATISTICAL ANALYSIS

SUPPLEMENTAL INFORMATION

Supplemental Information includes four figures and four tables and can be found with this article online at http://dx.doi.org/10.1016/j.cub.2017.04.055.

AUTHOR CONTRIBUTIONS

Conceptualization, J.K. and C.J.S.; Methodology/Resources, J.K., U.E.E., T.R.M., and C.J.S.; Investigation, J.K., J.P.L., E.L.H., D.M., A.P.D.Z., and C.J.S.; Formal Analysis, J.K., J.P.L., U.E.E., and C.J.S.; Writing – Original Draft/Visualization: J.K. and C.J.S.; Writing – Review & Editing, J.K., J.P.L., E.L.H., D.M., A.P.D.Z., T.R.M., U.E.E., and C.J.S.; Funding Acquisition/Supervision, J.K. and C.J.S.

ACKNOWLEDGMENTS

J.K. holds a Stevenson Junior Research Fellowship at University College, Oxford, and a Wellcome Trust Sir Henry Wellcome Postdoctoral Fellowship (204696/Z/16/Z). T.R.M. and C.J.S. both hold Wellcome Trust/Royal Society Henry Dale Fellowships (T.R.M. 104128/Z/14/Z; C.J.S. 102584/Z/13/Z). The work was additionally supported by the NIHR Oxford Biomedical Research Centre. Support for the 7-T scanner was provided by the Medical Research Council. The authors thank Bruce Gao for assistance with piloting the behavioral paradigm, Lewis Gaul for expertise in fitting psychophysics data, and Adam Berrington for advice on MRS acquisition and analysis. The schematic brain image in the graphical abstract was adapted from an original image by Patrick J. Lynch (https://coastfieldguides.com) licensed under Creative Commons Attribution 2.5 License 2006; no usage restrictions except please preserve creative credit. The schematic hand image used in the graphical abstract was adapted from an original image from http://www.clker.com/ licensed under Creative Commons CC0 1.0.

Received: November 15, 2016
Revised: March 7, 2017
Accepted: April 26, 2017
Published: May 25, 2017

REFERENCES

1. Thivierge, J.-P., and Marcus, G.F. (2007). The topographic brain: From neural connectivity to cognition. Trends Neurosci. *30*, 251–259.
2. Sillito, A.M. (1975). The contribution of inhibitory mechanisms to the receptive field properties of neurones in the striate cortex of the cat. J. Physiol. *250*, 305–329.
3. van Loon, A.M., Knapen, T., Scholte, H.S., St John-Saaltink, E., Donner, T.H., and Lamme, V.A.F. (2013). GABA shapes the dynamics of bistable perception. Curr. Biol. *23*, 823–827.
4. Puts, N.A.J., Edden, R.A.E., Evans, C.J., McGlone, F., and McGonigle, D.J. (2011). Regionally specific human GABA concentration correlates with tactile discrimination thresholds. J. Neurosci. *31*, 16556–16560.
5. Leventhal, A.G., Wang, Y., Pu, M., Zhou, Y., and Ma, Y. (2003). GABA and its agonists improved visual cortical function in senescent monkeys. Science *300*, 812–815.
6. Jasmin, L., Rabkin, S.D., Granato, A., Boudah, A., and Ohara, P.T. (2003). Analgesia and hyperalgesia from GABA-mediated modulation of the cerebral cortex. Nature *424*, 316–320.
7. Lunghi, C., Emir, U.E., Morrone, M.C., and Bridge, H. (2015). Short-term monocular deprivation alters GABA in the adult human visual cortex. Curr. Biol. *25*, 1496–1501.
8. Kolasinski, J., Makin, T.R., Jbabdi, S., Clare, S., Stagg, C.J., and Johansen-Berg, H. (2016). Investigating the stability of fine-grain digit somatotopy in individual human participants. J. Neurosci. *36*, 1113–1127.
9. Hittner, J.B., May, K., and Silver, N.C. (2003). A Monte Carlo evaluation of tests for comparing dependent correlations. J. Gen. Psychol. *130*, 149–168.
10. Nichols, T., Brett, M., Andersson, J., Wager, T., and Poline, J.-B. (2005). Valid conjunction inference with the minimum statistic. Neuroimage *25*, 653–660.
11. Hayes, A.F. (2013). Introduction to Mediation, Moderation, and Conditional Process Analysis (Guildford).
12. Semyanov, A., Walker, M.C., Kullmann, D.M., and Silver, R.A. (2004). Tonically active GABA A receptors: Modulating gain and maintaining the tone. Trends Neurosci. *27*, 262–269.
13. Glykys, J., and Mody, I. (2007). Activation of GABAA receptors: Views from outside the synaptic cleft. Neuron *56*, 763–770.
14. Semyanov, A., Walker, M.C., and Kullmann, D.M. (2003). GABA uptake regulates cortical excitability via cell type-specific tonic inhibition. Nat. Neurosci. *6*, 484–490.
15. Belelli, D., Harrison, N.L., Maguire, J., Macdonald, R.L., Walker, M.C., and Cope, D.W. (2009). Extrasynaptic GABAA receptors: Form, pharmacology, and function. J. Neurosci. *29*, 12757–12763.
16. Isaacson, J.S., and Scanziani, M. (2011). How inhibition shapes cortical activity. Neuron *72*, 231–243.
17. Kaas, J.H. (1997). Topographic maps are fundamental to sensory processing. Brain Res. Bull. *44*, 107–112.
18. Stagg, C.J., Bestmann, S., Constantinescu, A.O., Moreno, L.M., Allman, C., Mekle, R., Woolrich, M., Near, J., Johansen-Berg, H., and Rothwell, J.C. (2011). Relationship between physiological measures of excitability and levels of glutamate and GABA in the human motor cortex. J. Physiol. *589*, 5845–5855.
19. Zheng, M., Watanabe, K., and Hoshino, O. (2015). GABA diffusion across neuronal columns for efficient sensory tuning. Biol. Cybern. *109*, 493–503.
20. Farrant, M., and Nusser, Z. (2005). Variations on an inhibitory theme: Phasic and tonic activation of GABA(A) receptors. Nat. Rev. Neurosci. *6*, 215–229.
21. Yamada, J., Furukawa, T., Ueno, S., Yamamoto, S., and Fukuda, A. (2007). Molecular basis for the GABAA receptor-mediated tonic inhibition in rat somatosensory cortex. Cereb. Cortex *17*, 1782–1787.
22. Enna, S.J., Kuhar, M.J., and Snyder, S.H. (1975). Regional distribution of postsynaptic receptor binding for gamma-aminobutyric acid (GABA) in monkey brain. Brain Res. *93*, 168–174.
23. Harris, K.D., and Mrsic-Flogel, T.D. (2013). Cortical connectivity and sensory coding. Nature *503*, 51–58.
24. Li, Y.-T., Ibrahim, L.A., Liu, B.-H., Zhang, L.I., and Tao, H.W. (2013). Linear transformation of thalamocortical input by intracortical excitation. Nat. Neurosci. *16*, 1324–1330.

25. Li, L.-Y., Li, Y.-T., Zhou, M., Tao, H.W., and Zhang, L.I. (2013). Intracortical multiplication of thalamocortical signals in mouse auditory cortex. Nat. Neurosci. *16*, 1179–1181.

26. Lien, A.D., and Scanziani, M. (2013). Tuned thalamic excitation is amplified by visual cortical circuits. Nat. Neurosci. *16*, 1315–1323.

27. Takahashi, T., Kansaku, K., Wada, M., Shibuya, S., and Kitazawa, S. (2013). Neural correlates of tactile temporal-order judgment in humans: An fMRI study. Cereb. Cortex *23*, 1952–1964.

28. Davis, B., Christie, J., and Rorden, C. (2009). Temporal order judgments activate temporal parietal junction. J. Neurosci. *29*, 3182–3188.

29. Adhikari, B.M., Goshorn, E.S., Lamichhane, B., and Dhamala, M. (2013). Temporal-order judgment of audiovisual events involves network activity between parietal and prefrontal cortices. Brain Connect. *3*, 536–545.

30. Saal, H.P., and Bensmaia, S.J. (2014). Touch is a team effort: Interplay of submodalities in cutaneous sensibility. Trends Neurosci. *37*, 689–697.

31. Kim, S.S., Gomez-Ramirez, M., Thakur, P.H., and Hsiao, S.S. (2015). Multimodal interactions between proprioceptive and cutaneous signals in primary somatosensory cortex. Neuron *86*, 555–566.

32. Recanzone, G.H., Merzenich, M.M., and Jenkins, W.M. (1992). Frequency discrimination training engaging a restricted skin surface results in an emergence of a cutaneous response zone in cortical area 3a. J. Neurophysiol. *67*, 1057–1070.

33. London, B.M., and Miller, L.E. (2013). Responses of somatosensory area 2 neurons to actively and passively generated limb movements. J. Neurophysiol. *109*, 1505–1513.

34. Lee, S., Carvell, G.E., and Simons, D.J. (2008). Motor modulation of afferent somatosensory circuits. Nat. Neurosci. *11*, 1430–1438.

35. Lee, S., Kruglikov, I., Huang, Z.J., Fishell, G., and Rudy, B. (2013). A disinhibitory circuit mediates motor integration in the somatosensory cortex. Nat. Neurosci. *16*, 1662–1670.

36. van den Noort, J.C., van Beek, N., van der Kraan, T., Veeger, D.H.E.J., Stegeman, D.F., Veltink, P.H., and Maas, H. (2016). Variable and asymmetric range of enslaving: Fingers can act independently over small range of flexion. PLoS ONE *11*, e0168636.

37. Oldfield, R.C. (1971). The assessment and analysis of handedness: The Edinburgh inventory. Neuropsychologia *9*, 97–113.

38. Lemke, C., Hess, A., Clare, S., Bachtiar, V., Stagg, C., Jezzard, P., and Emir, U. (2015). Two-voxel spectroscopy with dynamic B0 shimming and flip angle adjustment at 7 T in the human motor cortex. NMR Biomed. *28*, 852–860.

39. Scheenen, T.W.J., Klomp, D.W.J., Wijnen, J.P., and Heerschap, A. (2008). Short echo time 1H-MRSI of the human brain at 3T with minimal chemical shift displacement errors using adiabatic refocusing pulses. Magn. Reson. Med. *59*, 1–6.

40. Tkác, I., Starcuk, Z., Choi, I.Y., and Gruetter, R. (1999). In vivo 1H NMR spectroscopy of rat brain at 1 ms echo time. Magn. Reson. Med. *41*, 649–656.

41. Kataoka, K., Nakamura, Y., Hassler, R., Bak, I.J., and Kim, J.S. (1975). Activity of L-glutamic acid decarboxylase in different regions of baboon brain. Folia Psychiatr. Neurol. Jpn. *29*, 361–370.

42. Jenkinson, M., Beckmann, C.F., Behrens, T.E.J., Woolrich, M.W., and Smith, S.M. (2012). FSL. Neuroimage *62*, 782–790.

43. Jenkinson, M., Bannister, P., Brady, M., and Smith, S. (2002). Improved optimization for the robust and accurate linear registration and motion correction of brain images. Neuroimage *17*, 825–841.

44. Smith, S.M. (2002). Fast robust automated brain extraction. Hum. Brain Mapp. *17*, 143–155.

45. Cabanes, E., Confort-Gouny, S., Le Fur, Y., Simond, G., and Cozzone, P.J. (2001). Optimization of residual water signal removal by HLSVD on simulated short echo time proton MR spectra of the human brain. J. Magn. Reson. *150*, 116–125.

46. Provencher, S.W. (2001). Automatic quantitation of localized in vivo1H spectra with LCModel *14*, 260–264.

47. Zhang, Y., Brady, M., and Smith, S. (2001). Segmentation of brain MR images through a hidden Markov random field model and the expectation-maximization algorithm. IEEE Trans. Med. Imaging *20*, 45–57.

48. Shore, D.I., Gray, K., Spry, E., and Spence, C. (2005). Spatial modulation of tactile temporal-order judgments. Perception *34*, 1251–1262.

49. Azañón, E., Stenner, M.-P., Cardini, F., and Haggard, P. (2015). Dynamic tuning of tactile localization to body posture. Curr. Biol. *25*, 512–517.

50. Preacher, K.J., and Kelley, K. (2011). Effect size measures for mediation models: Quantitative strategies for communicating indirect effects. Psychol. Methods *16*, 93–115.

STAR★METHODS

KEY RESOURCES TABLE

REAGENT or RESOURCE	SOURCE	IDENTIFIER
Software and Algorithms		
MATLAB 2014b	The MathWorks, Natick, MA, USA	https://uk.mathworks.com/products/new_products/release2014b.html
JMP12	SAS Institute, Cary, NC, USA	https://www.jmp.com
SPSS 22	IBM Corporation, Armonk, NY, USA	https://www.ibm.com/analytics/us/en/technology/spss/
FMRIB Software Library (FSL) 5.0	Oxford Centre for fMRI of the Brain (FMRIB), Oxford, UK.	https://fsl.fmrib.ox.ac.uk/fsl/fslwiki

CONTACT FOR RESOURCE SHARING

Further information and requests for resources should be directed to and will be fulfilled by the Lead Contact, James Kolasinski (james.kolasinski@ndcn.ox.ac.uk).

EXPERIMENTAL SUBJECT DETAILS

All data were acquired in accordance with local central university research ethics committee approval (University of Oxford MS-IDREC-C2-2015-014/C1-2014-100). All participants provided written informed consent, were right handed according to the Edinburgh Handedness Inventory [37], had no history of neurological or psychiatric illness, and met local MRI safety criteria.

Experiment one collected magnetic resonance spectroscopy (MRS) measurement and fMRI-derived cortical tuning measurements in primary somatosensory cortex from 22 participants (10 female, mean age: 22.86 ± 3.81), 11 of whom also undertook at tactile temporal order judgment task to derive measures of perceptual acuity (5 female, mean age: 21.73 ± 2.97).

Experiment two was subsequently undertaken to replicate the results observed in experiment one, as well to acquire additional MRS measurements from bilateral primary visual cortex as an anatomical control. An independent cohort of 11 participants was recruited to this experiment (5 female, mean age: 21.36 ± 2.67), all of whom also undertook the same MR measurements and tests of perceptual acuity as were used in experiment one.

Full information on participant demographics is provided in Table S1.

METHOD DETAILS

MR acquisition

MR data were acquired using a Siemens 7T Magnetom system with a 32-channel head coil. Dielectric pads (barium titanate: 11 × 11cm and 5mm) were positioned to increase B1 efficiency in the regions of interest for MRS acquisition [38]. B1 efficiency was mapped using actual flip angle imaging (AFI) (FOV 240x240, TR1 6 ms, TR2: 30 ms, TE 2.58 ms, slice thickness 2.5 mm, non-selective flip angle 60°). Dielectric pads were re-positioned to achieve optimal B1 efficiency when necessary.

Blood oxygenation level dependent (BOLD) fMRI was acquired using a T2*-weighted multislice gradient echo planar imaging (EPI), with true axial slices centered on the left anatomical hand knob in the z axis (TR 1,500 ms, TE 25 ms, slice thickness 1.2 mm, in-plane resolution 1.2 × 1.2 mm, 22 axial slices, GRAPPA factor = 2).

Structural MRI data were acquired to aid MRS voxel placement using a magnetization prepared rapid acquisition gradient echo (MPRAGE) sequence (TR 2200 ms, TE 2.82 ms, slice thickness 1.0 mm, in-plane resolution 1.0 × 1.0 mm, GRAPPA factor = 2).

MRS data were acquired using a semi-LASER sequence [39] (64 averages, TR 5000ms, TE 36ms, Voxel size 20x20x20mm) using VAPOR (variable power RF pulses with optimized relaxation delays) water suppression [40]. The voxel of interest (VOI) was manually positioned in the left sensorimotor cortex, covering the whole hand knob structure (experiment one and two), or bilateral primary visual cortex, over the calcarine sulcus of occipital cortex (experiment two), in both cases avoiding contact with the dura to minimize significant macromolecule contamination. Measurements from the sensorimotor voxel provided a robust measure of somatosensory GABA concentrations given the known concordance of GABA levels across motor and somatosensory cortex [22, 41].

fMRI task

fMRI data were acquired during an active motor task in which participants made visually cued movements of individual fingers of the right hand (2: index, 3: middle, 4: ring, 5: little) in the form of button presses made on an MRI-compatible button box (manufactured in-house) resting on the right thigh. Instructions were projected onto a screen inside the scanner bore, in the form of four white circles,

representing the four fingers. The circles flashed individually at a frequency of 1 Hz, instructing presses of that finger at this rate. The task involved continuous movement, with 8 s blocks involving each finger. The forward version of this task cycled from finger 2 to finger 5 inclusive, with the resulting 32 s cycle repeated eight times. The backward version of the task was identical in duration, but cycled from finger 5 to finger 2 inclusive. Total fMRI acquisition was 8 min and 50 s. This paradigm has been applied and validated previously [8].

fMRI analysis

Travelling wave analysis

fMRI data were processed using the FMRIB Software Library [42], including motion correction with MCFLIRT [43], brain extraction using BET [44], and high pass temporal filtering (100 s cut-off). All fMRI analysis was conducted in the individual subject fMRI space, with no co-registration across subjects or to a standard space. No smoothing of the data was performed.

Traveling wave analysis of the continuous finger movements involved in the fMRI task have been outlined previously [8]. This approach applies cross-correlation of the task data against a number of iteratively time-shifted models, in order to model voxel-wise tuning curves, which show the point in the movement cycle at which each voxel responds maximally. The model applied was a gamma-convolved boxcar: 8 s 'on' and 24 s 'off', repeated eight times, to mirror a single block of movement of one digit. This model was shifted sufficient times to model peaks across the entire digit cycle (forward: finger 2 – 5, backward: finger 5 – 2). The result was a four-dimensional voxel-wise map for both the forward and backward task variant, in which each voxel contained a cortical tuning profile of activity during the cycle of digit movement, expressed in *r*-values (Figure 1B: gray).

Cortical tuning metric

For the forward and backward task variants, the cortical activity profile in each voxel was used to fit a sinusoidal function (Figure 1B: green) using MATLAB 2014b (The MathWorks, Natick, MA, USA), with the following general equation and a least-squares cost function:

$$y = a\,\sin(\omega x - \varphi) + B.$$

This process yielded voxel-wise maps of the amplitude (a), period (ω) of the cortical activity profile. These maps were averaged across the forward and backward task variant. The amplitude maps were used to generate a region-of-interest (ROI) corresponding to the region of peak activation in the task: this was calculated using voxel-wise false discovery rate (FDR) thresholding of the amplitude map after an *r*-to-*Z* transformation ($\alpha = 0.01$), yielding a region corresponding directly to the anatomical hand knob for each participant (Figure 1A). To calculate a measure of cortical tuning across the hand knob, the period of each voxel underlying the ROI was averaged, and expressed as the inverse (1/period) to yield an intuitive metric. Greater magnitude of this metric reflects greater cortical tuning: a more selective activation during the cycle of movement (Figure 1C).

fMRI behavioural task analysis

For the forward and backward variants of the fMRI task, measures of button press rate and button press accuracy were quantified. Button press rate was quantified as the interval between two button presses, excluding any statistical outliers (+/− 2 standard deviations from the mean rate). Button press accuracy was quantified for each of the 256 button presses in each task. An accurate response was defined as a button press, which began within 400 ms of the stimulus signal instructing each button press.

MRS analysis

Raw data were corrected using the unsuppressed water signal from the same VOI, eddy current correction, a zero-order phasing of array coil spectra. Residual water signal was removed using Hankel-Lanczos singular value decomposition [45]. Neurochemicals within the chemical shift range 0.5 to 4.2 ppm were quantified with LCModel analysis [46]. The exclusion criteria for data were as follows: Cramér-Rao bounds > 50%, water linewidths at full width at half maximum (FWHM) > 15 Hz, and (iii) SNR < 40. In experiment two, the V1 spectrum from one participant was excluded on the basis of these criteria. Correlation analysis with a lower Cramér-Rao bounds threshold (< 30%) are presented in Figure S3. Good spectral separation was achieved for GABA, indicated by no strong correlations (> +/− 0.3 in magnitude) between absolute concentration GABA and any other metabolite (Figure S4).

FMRIB's Automated Segmentation Tool (FAST) [47] was used to calculate the contributions of gray matter (GM) and white matter (WM) to the VOI from the T1-weighted MPRAGE scan. GABA and glutamate peaks were corrected for the proportion of GM:

$$\frac{[Absolute\ GABA]}{\dfrac{[GM]}{[GM]+[WM]+[CSF]}}.$$

Total creatine (including phosphocreatine) peaks were corrected for the proportion of total brain volume:

$$\frac{[Absolute\ Cr]+[Absolute\ PCr]}{\dfrac{[GM]+[WM]}{[GM]+[WM]+[CSF]}}.$$

GABA and glutamate values are reported as a ratio to total creatine.

Behavioral data acquisition

A subset of 11 participants from experiment one, and all participants in experiment two (n = 11) performed a temporal order judgment task involving vibrotactile stimuli presented to the ventral distal pads of the right index and middle finger; a commonly used paradigm across perceptual studies of somatosensation [48, 49]. Participants performed the task in a testing lab, sitting in a static chair placed a fixed distance from a table, on which their arms rested uncrossed. Each of their feet rested on a simple binary-state foot pedal. The right hand was positioned in the midline on a sheet of polyurethane foam, in which two miniature electromagnetic solenoid-type stimulators were embedded (Tactors, Chris Dancer Design, St Helens, UK; 20 ms stimulation, 200 Hz sinusoidal pattern, > 10 times threshold amplitude), such that ventral distal pad of the index and middle fingers were directly opposed to one stimulator. The relative positions of the stimulator within the foam could be adjusted to accommodate anatomical variability. The view of the right hand was occluded, and participants listened to pink noise sufficient to mask auditory cues from the stimuli. Participants were instructed to fixate on a central fixation cross present on the screen at all times; monitoring was in place to ensure participants' eyes remained open at all times.

A two alternative forced choice task was delivered, with instructions presented on an all-in-one desktop machine, positioned at eye level at a distance of 56cm. Participants performed 192 trials, during each of which a single stimulus was delivered to each of the two fingers. For each trial, participants were asked to judge which stimulus came first, by pressing either the left or the right foot pedal. Participants were cued with the word "Ready" on the screen to signify an imminent trial, "Response" to prompt a judgment, and "No response" in cases where no judgment was made within two seconds.

The interstimulus intervals (ISIs) used were +350, −350, +250, −250, +150, −150, +100, −100, +60, −60, +30 and −30ms, with a positive ISI denoting the middle finger was stimulated first. Each ISI was presented in 16 trials, in a randomly assigned order, consistent across participants.

Behavioral data analysis

The proportion of right-first responses for each ISI was fitted to a logistic function (Figure 1E) using MATLAB and Statistics Toolbox Release 2014b (The MathWorks, Natick, MA, USA). All data far exceeded our quality of fit criteria ($r^2 > 0.4$; actual range: 0.829 – 0.999). For each participant, a value of perceptual acuity was calculated as the just noticeable different (JND): half the difference between the two ISIs that give a "middle finger first" judgement in 75% and 25% of trials: smaller values of JND therefore reflect higher perceptual acuity.

Reproducibility measures

The reproducibility of the cortical tuning metric and JND values was calculated from data collected in two independent cohorts, each comprising sixteen participants. JND data were collected at two time points, separated by an interval of one week. Cortical tuning metrics were calculated from fMRI data acquired as two time points, separated by an interval of four weeks.

QUANTIFICATION AND STATISTICAL ANALYSIS

Statistical analyses and graphing were undertaken using JMP (Version 12.0, SAS Institute, Cary, NC, USA) and Statistics Package for the Social Sciences (SPSS, Version 22.0, IBM Corporation, Armork, NY, USA). All statistical tests presented herein are two-tailed, and have been performed on the assumption of normality (Shapiro-Wilk confirmed).

Data from experiment 1 (n = 11) and 2 (n = 11) were analyzed using Pearson correlation coefficients. Each experiment considered the relationships between MRS, fMRI and tactile psychophysics data. In experiment 1, fMRI and MRS data were also available for an additional 11 participants; correlation coefficients are therefore presented for this extended cohort (n = 22), in addition to the original cohort (n = 11). Correlation metrics were compared using Hittner's test. Correlation statistics are presented in the results and Figures 2 and 3.

We applied a conjunction test over all predicted correlations and an omnibus test over all non-predicted correlations [10, 12, 13]. In this stringent analysis, the compound null hypothesis is disproved only if all predicted correlations are significant in the absence of significant unpredicted correlations. The conjunction test therefore considers the maximum p value across all predicted correlations, while the omnibus test considers the minimum p value from all non-predicted correlations, the combination of which provide evidence to reject the null hypothesis. This liberal test offers good power to detect any possible correlations that deviate from the expected hypothesis, and to account for multiple correlation analyses undertaken. The results of the conjunction and omnibus tests are presented in the results.

In order to more fully explore the observed correlations between GABA measures, fMRI-derived cortical tuning measures, and tactile perceptual acuity, a mediation analysis was conducted across the full-datasets collected across experiments 1 and 2 (n = 22). Mediation was conducted using regression with bootstrapping to ascertain whether cortical tuning accounts for the link between cortical inhibition and perceptual abilities. This analysis was conducted using the PROCESS macro for SPSS [11]. The Sobel test and Preacher and Kelley's κ^2 to determine the significance and strength of the effect respectively [50]. The results of this analysis can be found in Figure 4 and Table S4.

Reproducibility measures were calculated in two cohorts independent of experiments 1 and 2 using an intraclass correlation coefficient ICC (two-way random effects model with absolute agreement).

Current Biology, Volume *27*

Supplemental Information

A Mechanistic Link from GABA

to Cortical Architecture and Perception

James Kolasinski, John P. Logan, Emily L. Hinson, Daniel Manners, Amir P. Divanbeighi Zand, Tamar R. Makin, Uzay E. Emir, and Charlotte J. Stagg

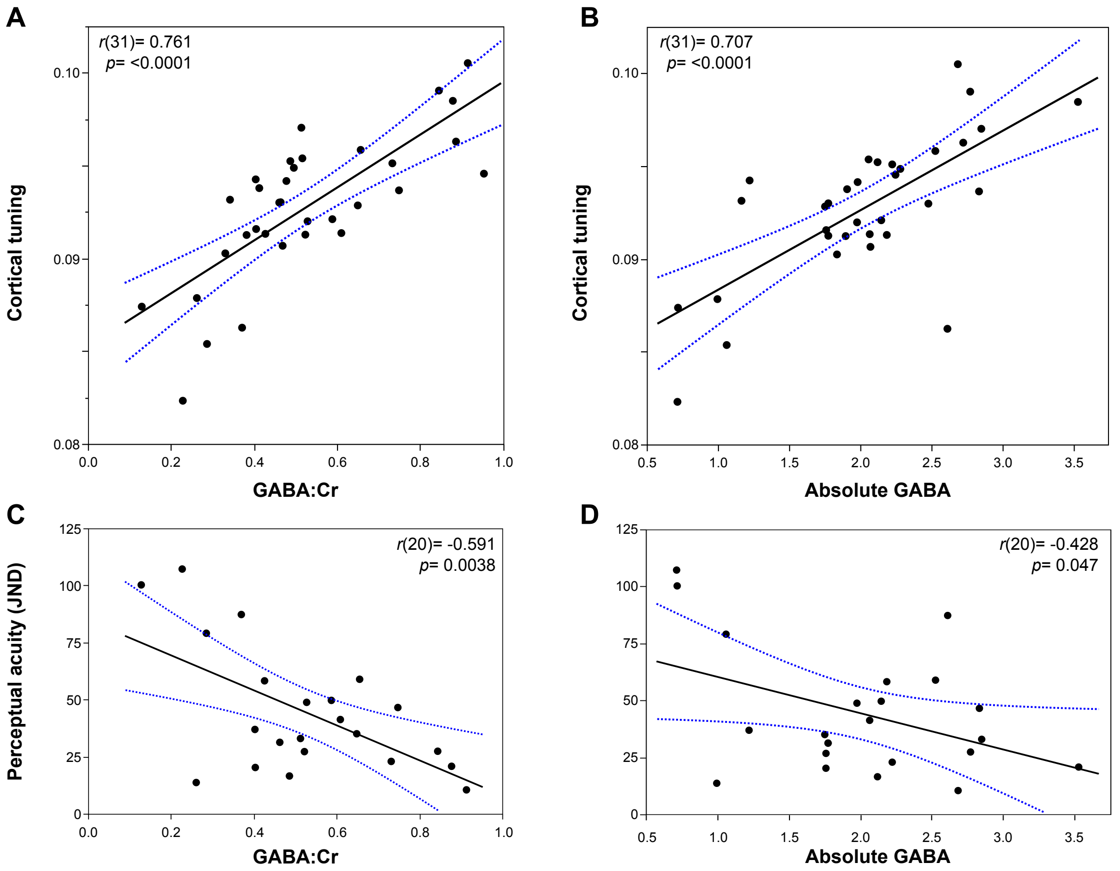

Figure S1. Correlations with GABA:Cr persist for raw GABA concentrations. Related to Figures 2 & 3. The observed patterns of correlation between normalised GABA:Cr values and both cortical tuning (A) and perceptual acuity (C) were also observed for values of absolute GABA quantified directly from the LCmodel MRS data analysis (B/D). In this combined cohort, the relationship between cortical tuning and tactile perceptual acuity was also still observed: r(20)=-0.734 p=0.0001 (not shown).

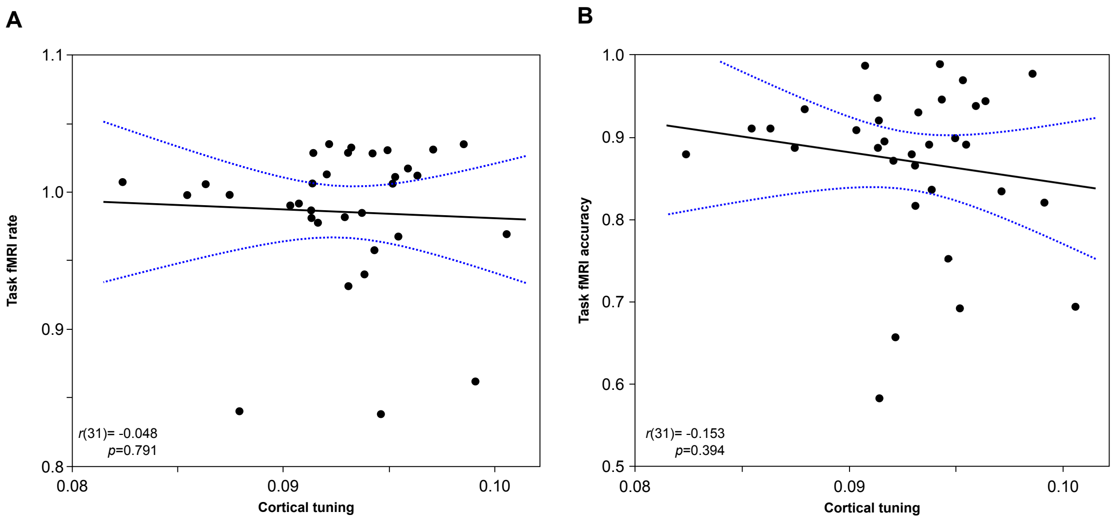

Figure S2. Cortical tuning metrics calculated in S1 show no relationship with motor performance during fMRI task. Related to Figures 2 & 3. Neither task fMRI motor rate (A: button presses per second) nor accuracy (B: percentage correct button presses) correlated with the magnitude of the cortical tuning metric across participants.

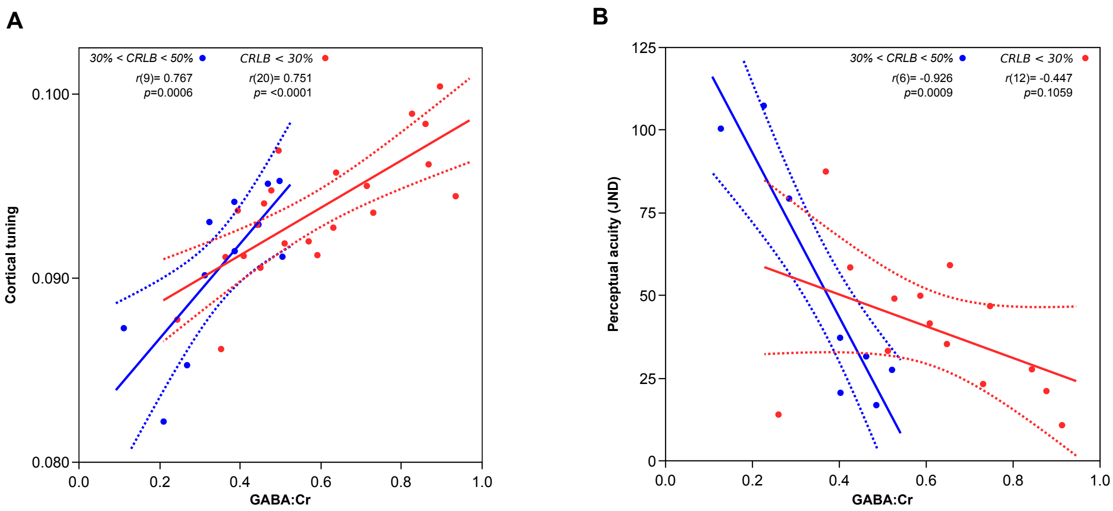

Figure S3. Analysis of correlations of GABAergic tone metrics assessed by MRS model fit. Related to Figures 2 & 3. Correlations between GABA:Cr and both cortical tuning and tactile perceptual acuity are present across the range of CRLB thresholds applied to fitted MRS data.

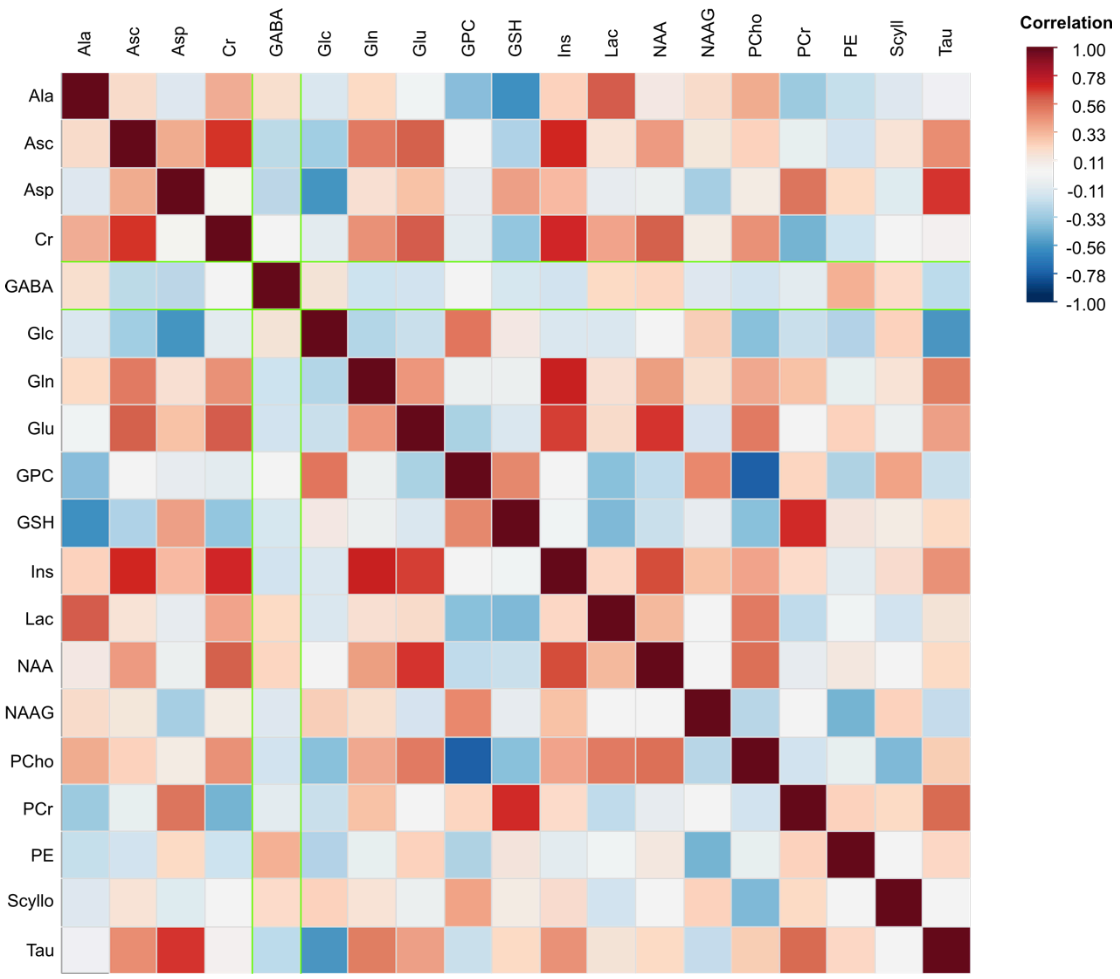

Figure S4. Cross-correlation of metabolites quantified by MRS in all participants. Related to Figures 2 & 3. Pearson's correlation coefficient shows no correlations between absolute concentrations of GABA and other metabolites (green column/row) greater in magnitude than +/- 0.35. This result indicates good spectral separation of GABA.

		ID	Age	Gender	Handedness
**Experiment one**	*MR only*	1-001	23	F	R
		1-002	26	F	R
		1-003	28	F	R
		1-004	25	M	R
		1-005	20	M	R
		1-006	20	M	R
		1-007	19	M	R
		1-008	22	M	R
		1-009	32	F	R
		1-010	29	M	R
		1-011	20	F	R
	MR + behaviour	1-012	21	M	R
		1-013	20	M	R
		1-014	30	F	R
		1-015	21	M	R
		1-016	24	F	R
		1-017	21	M	R
		1-018	20	M	R
		1-019	20	M	R
		1-020	20	F	R
		1-021	21	F	R
		1-022	21	F	R
**Experiment two**	*MR + behaviour*	2-001	20	F	R
		2-002	26	M	R
		2-003	27	F	R
		2-004	20	F	R
		2-005	20	M	R
		2-006	22	M	R
		2-007	20	M	R
		2-008	20	F	R
		2-009	19	F	R
		2-010	21	M	R
		2-011	20	M	R

Table S1. Demographic information for participants recruited to fMRI and behavioural cohorts. Related to Figure 1. F: female, M: Male, R: right handed, MR: magnetic resonance.

ID	Tuning: time point 1	Tuning: time point 2
3-001	0.089074	0.090526
3-002	0.093360	0.090194
3-003	0.091256	0.090504
3-004	0.091828	0.090704
3-005	0.088730	0.091726
3-006	0.091622	0.088814
3-007	0.088088	0.087834
3-008	0.086516	0.087832
3-009	0.085434	0.083752
3-010	0.089918	0.089202
3-011	0.085746	0.084584
3-012	0.083318	0.084836
3-013	0.089762	0.091070
3-014	0.090726	0.090726
3-015	0.088142	0.088264
3-016	0.088274	0.088102

Table S2. Test-Retest reliability of cortical tuning metric. Related to Figures 2 & 3. Data collected during the same task fMRI paradigm in a cohort independent of the current study during two scan sessions, separated by 4 weeks. A high degree of internal consistency was observed between the JND measurements derived on separate days. The single measures intraclass correlation (ICC) was 0.802 (95% confidence interval: 0.520 - 0.926). ICC calculated using a two-way random effects model with absolute agreement.

ID	JND: time point 1	JND: time point 2
4-001	23.591	23.566
4-002	57.429	64.634
4-003	30.486	40.327
4-004	39.912	37.693
4-005	45.139	48.660
4-006	31.440	39.458
4-007	25.683	39.682
4-008	32.320	41.779
4-009	23.217	14.694
4-010	27.674	24.719
4-011	46.802	40.453
4-012	35.319	43.581
4-013	49.924	46.385
4-014	106.719	77.447
4-015	10.774	23.359
4-016	45.879	53.090

Table S3. Test-Retest reliability of JND measures. Related to Figures 2 & 3. Data collected during a temporal order judgment task in a cohort independent of the current study during two behavioural testing sessions, separated by 1 week. A high degree of internal consistency was observed between the JND measurements derived on separate days. The single measures intraclass correlation (ICC) was 0.842 (95% confidence interval: 0.610 - 0.942). ICC calculated using a two-way random effects model with absolute agreement. JND: Just Noticeable Difference

		Consequent						
		M (Tuning)				Y (JND)		
Antecedent		Coeff.	*SE*	*p*		Coeff.	*SE*	*p*
X (GABA)	*a*	0.1807	0.0259	<0.001	*c'*	-0.0118	0.0373	0.7552
M (Tuning)		-	-	-	*b*	0.4893	0.1739	0.0110
Constant	i_1	0.8298	0.0147	<0.001	i_2	-0.2909	0.1447	0.0588
		R^2= 0.7086<br>$F_{(1,20)}$=48.6356 p<0.001				R^2= 0.5409<br>$F_{(2,19)}$=11.1908 p<0.001		
		Total effect (*c*)<br>Coeff.= 0.0766 SE= 0.0234<br>p=0.0038<br><br>Direct effect (*c'*)<br>Coeff.= -0.0118 SE= 0.0373<br>p=0.7552				Indirect effect (ab)<br>Coeff.= 0.0884 SE=0.0359<br><br>Preacher and Kelley κ^2<br>κ^2=0.46 SE=0.1479		

Table S4. Summary statistics from mediation analysis. Related to Figure 4.
